# Keratinocyte Motility Is Affected by UVA Radiation—A Comparison between Normal and Dysplastic Cells

**DOI:** 10.3390/ijms19061700

**Published:** 2018-06-07

**Authors:** Cristina M. Niculiţe, Marina T. Nechifor, Andreea O. Urs, Laura Olariu, Laura C. Ceafalan, Mircea Leabu

**Affiliations:** 1 Victor Babeș National Institute of Pathology, 99-101, Splaiul Independentei, 050096 Bucharest, Romania; cristina_niculite@yahoo.com (C.M.N.); aurs.bv@gmail.com (A.O.U.); lauraceafalan@yahoo.com (L.C.C.); 2 Department of Morphological Sciences, University of Medicine and Pharmacy Carol Davila, 8, Blvd. Eroilor Sanitari, 050474 Bucharest, Romania; 3 Department of Anatomy, Animal Physiology and Biophysics, Faculty of Biology, University of Bucharest, 91-95, Splaiul Independentei, 050095 Bucharest, Romania; nemar_59@yahoo.com; 4 SC Biotehnos SA, 3-5, Gorunului Street, 075100 Otopeni, Romania; lolariu@biotehnos.com

**Keywords:** cell motility, keratinocyte, dysplastic cell, wound healing, actin cytoskeleton, focal contacts

## Abstract

UVA radiation induces multiple and complex changes in the skin, affecting epidermal cell behavior. This study reports the effects of UVA exposure on normal (HaCaT) and dysplastic (DOK) keratinocytes. The adherence, spreading and proliferation were investigated by time-lapse measurement of cell layer impedance on different matrix proteins. Prior to UVA exposure, the time required for adherence and spreading did not differ significantly for HaCaT and DOK cells, while spreading areas were larger for HaCaT cells. Under UVA exposure, HaCaT and DOK cells behavior differed in terms of movement and proliferation. The cells’ ability to cover the denuded surface and individual cell trajectories were recorded by time-lapse videomicroscopy, during wound healing experiments. Dysplastic keratinocytes showed more sensitivity to UVA, exhibiting transient deficiencies in directionality of movement and a delay in re-coating the denuded area. The actin cytoskeleton displayed a cortical organization immediately after irradiation, in both cell lines, similar to mock-irradiated cells. Post-irradiation, DOK cells displayed a better organization of stress fibers, persistent filopodia, and new, stronger focal contacts. In conclusion, after UVA exposure HaCaT and DOK cells showed a different behavior in terms of adherence, spreading, motility, proliferation, and actin cytoskeleton dynamics, with the dyplastic keratinocytes being more sensitive.

## 1. Introduction

Ultraviolet type A (UVA) radiation (320–400 nm) represents the main component of the ultraviolet (UV) solar spectrum reaching the Earth’s surface and acting as a stress factor on skin [1,2]. In addition to solar UVA radiation, human skin might be exposed to relatively high doses of UVA during various therapeutic [3] and cosmetic procedures [4]. UVA irradiation induces oxidative stress and activates complex signaling pathways which are involved in fundamental cellular events in the skin, including adhesion, proliferation, differentiation, senescence, malignant transformation and cell death [5,6,7,8]. UVA photons deeply penetrate through the skin [9]. Therefore, UVA can disturb epidermal intercellular adhesion [10] and cell-to-cell communication mediated by gap junctions as shown in primary keratinocytes [11]. It may be presumed that UVA exposure also affects the attachment of keratinocytes to the basement membrane, which might disturb their motility.

Migration of skin cells plays an important role in a variety of physio-pathological processes, such as wound healing [12], tumor invasion and metastasis [13,14]. Cell migration is a multistep process depending on the extracellular matrix composition and involving changes in the cytoskeleton organization, cell-to-cell/cell-substrate adhesion, and other specific changes of cellular components associated with the dynamic reshaping of the migrating cells [15,16,17]. Despite the fact that the control of cell migration by various biochemical and biophysical factors is well documented [18,19,20,21,22,23], to our knowledge, a precise investigation of UVA effects on skin cell migration is unreported.

In this study, we aimed to explore the effect of UVA exposure on the behavior of normal keratinocyte of adult human origin (HaCaT) and dysplastic oral keratinocytes (DOK). We have investigated the effects on the adherence, spreading, proliferation and motility of keratinocytes seeded on different extracellular matrix proteins by cell layer impedance measurements and by time-lapse videomicroscopy in a wound healing model. Our results proved that HaCaT and DOK cells have behaved differently after UVA exposure, with the most notable change being the loss in the directionality of movement for the dysplastic cells, accompanied by altered dynamics of actin cytoskeleton and focal contacts.

## 2. Results

### 2.1. Effects of UVA Radiation on Cell Attachment, Spreading and Proliferation

HaCaT and DOK cell behavior before and after UVA irradiation was assessed by time-lapse monitoring of the cell layer impedance, on surfaces coated with matrix proteins at three different concentrations (2.5, 5.0 and 10 µg/mL). The measured impedance signal was proportional to the attachment and spreading of the cells on surfaces coated with matrix proteins. The recorded adhesion data (cell index) allowed the evaluation of spreading areas and the time periods required for attachment, as well as the start of proliferation.

#### 2.1.1. Cell Behavior before UVA Exposure

**Figure 1:**
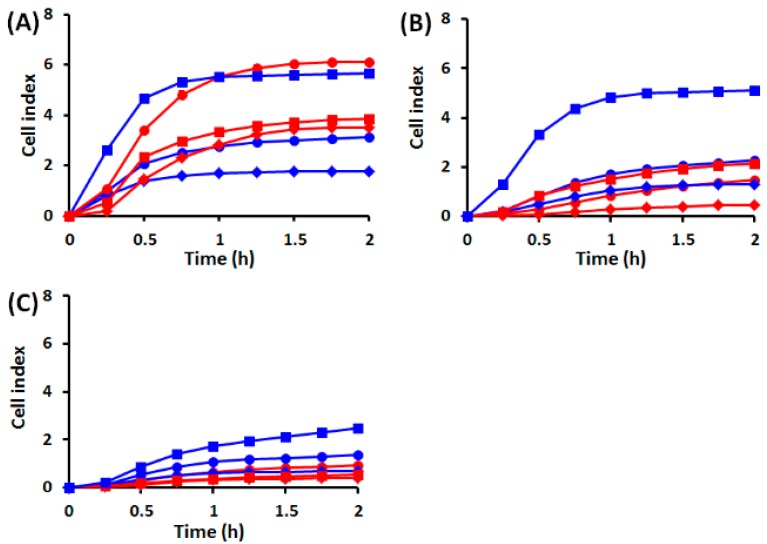
Kinetics of cell adherence and spreading for HaCaT (red plots) and DOK (blue plots) cells, on various matrix proteins, at different concentrations. (**A**) Collagen; (**B**) fibronectin; (**C**) laminin; diamonds—2.5 µg/mL, circles—5 µg/mL, squares—10 µg/mL.

Our results indicate that before UVA exposure, the time required for the attachment and spreading of HaCaT and DOK cells on coated surfaces did not differ significantly, being about 1 h, regardless of the concentration of matrix protein. However, adherence and spreading, as suggested by cell index, were considerably larger for dysplastic cells than for normal ones, except for HaCaT cells on collagen that showed biphasic behavior (Figure 1). Both cell types showed a lower adherence and spreading on fibronectin (Figure 1B) and laminin (Figure 1C), as compared to collagen (Figure 1A). Moreover, on both fibronectin and laminin normal keratinocyte adherence and spreading were not significantly dependent on the matrix protein concentration, as compared with dysplastic cells. Noteworthy, on collagen, both cell types showed the largest adherence and spreading and a more obvious dependence on matrix protein concentration. However, HaCaT cell adherence and spreading were not significantly different at low and high concentrations, but reached the highest level at 5 µg/mL collagen. Conversely, DOK cell adherence and spreading were directly proportional with the collagen concentration. These results guided us to use 5 µg/mL collagen-coated surfaces for the next sets of experiments.

#### 2.1.2. Cell Behavior after UVA Exposure

**Figure 2:**
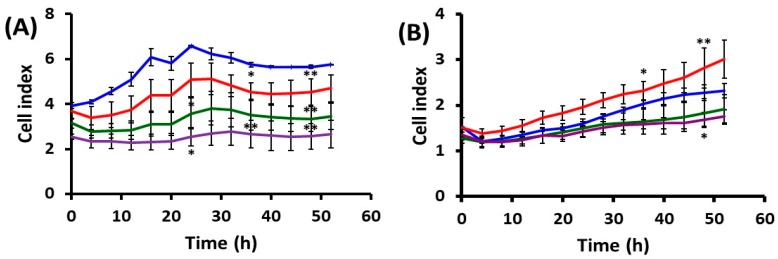
Kinetics of cell adherence, spreading and proliferation for keratinocytes after UVA irradiation on 5 µg/mL collagen. (**A**) HaCaT; (**B**) DOK; blue plots—mock-irradiated cells; red plots—15 min irradiation; green plots—30 min irradiation; purple plots—60 min irradiation. * *p* < 0.05, ** *p* < 0.01.

The experiments monitored the effect of three environmentally relevant doses of irradiation (accumulated during 15, 30 or 60 min experimental exposure). UVA exposure affected the two cell lines differently in terms of spreading area, suggesting that HaCaT and DOK cells’ ability to adhere and spread was specifically affected by UVA irradiation, as compared with the mock-irradiated cells (Figure 2). During the exposure, HaCaT cells showed a reduction in the spreading area that was directly proportional with the UVA dose (Figure 2A). Surprisingly, for dysplastic keratinocytes the spreading area increased under the low UVA dose, which accumulated during 15 min exposure (Figure 2B).

Furthermore, UVA exposure differentially affected the proliferation of normal and dysplastic keratinocytes. As compared with mock-irradiated cells, both a lower rate and a specific delay in proliferation were recorded at distinct UVA doses for normal keratinocytes (Figure 2A). Dysplastic cells were less affected by UVA, regardless of the dose. However, at the lowest UVA dose the cell index was constantly higher and continuously increasing as compared with the control. The 30 and 60 min exposure seems to inhibit cell proliferation, despite the fact that time-lapse videomicroscopy mitoses was observed.

### 2.2. Effects of UVA Radiation on Cell Motility during In Vitro Wound Healing

The ability of cells to actively locomote is very important in the process of wound healing. Our aim was to assess whether UVA exposure affects cell motility. Using time-lapse videomicroscopy and an associated analysis of digital image time series, we recorded the collective migration and the individual cell trajectories during the coverage of the scratched surface area, according to the in vitro wound healing assay.

#### 2.2.1. Collective Cell Migration

**Figure 3:**
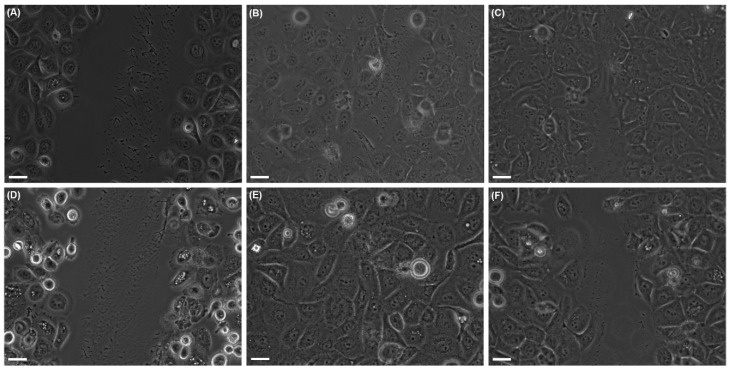
Effects of UVA irradiation on the ability of keratinocytes to cover the scratched area, in wound-healing experiments. (**A**–**C**)—HaCaT; (**D**–**F**)—DOK. (**A**,**D**) Mock-irradiated cells right after scratching; (**B**,**E**) mock-irradiated cells at 6 h after scratching; (**C**,**F**) cells irradiated for 30 min, at 6 h after UVA exposure. Scale bars represent 25 µm.

**Figure 4:**
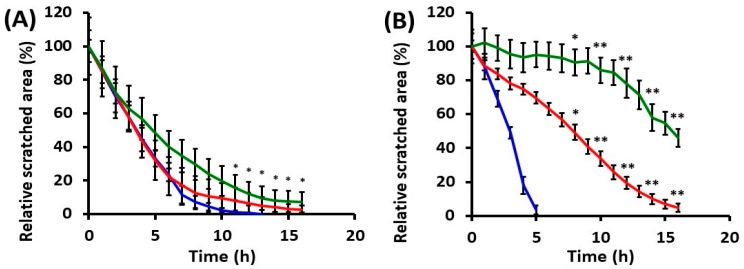
Effects of UVA exposure on the motility of keratinocytes, in terms of their ability to re-cover the scratched area. (**A**) HaCaT; (**B**) DOK. Blue plots—mock-irradiated cells; red plots—cells after 30 min irradiation; green plots—cells after 60 min irradiation. * *p* < 0.05, ** *p* < 0.01.

Cell behavior and ability to re-coat the denuded surface were monitored for both mock-irradiated and UVA-irradiated cells. HaCaT and DOK cells were differently affected by UVA exposure, the time needed for the scratch-wound closure being obviously different (Figure 3 and Figure 4). For HaCaT cells, the ability to re-coat the denuded area was not dramatically affected by UVA exposure, although dose-dependent cell behavior was noted (Figure 4A). The irradiated dysplastic cells proved to need much longer time periods for wound closure in both irradiation conditions, as compared with mock-irradiated cells (Figure 4B). Thus, after 30 min irradiation, DOK needed thrice as long time (~16 h) to cover the denuded surface, in comparison with the mock-irradiated DOK (~5 h), while the effect was even more striking following the high dose of UVA radiation (Figure 4B). Moreover, our results showed that dysplastic keratinocyte motility was higher than that of normal cells in the absence of UVA exposure.

#### 2.2.2. Individual Cell Trajectories

**Figure 5:**
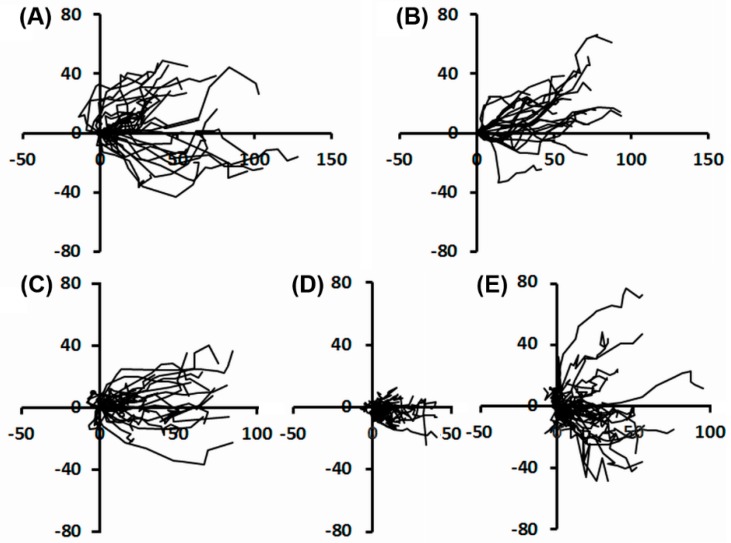
Effects of UVA exposure on the motile ability of keratinocytes, in terms of their directionality of movement. (**A**) Trajectories of mock-irradiated HaCaT cells during the first 5 h after scratching; (**B**) trajectories of HaCaT cells irradiated for 30 min, during the first 5 h after scratching; (**C**) trajectories of mock-irradiated DOK cells during the first 5 h after scratching; (**D**) trajectories of DOK cells irradiated for 30 min, during the first 5 h after scratching; (**E**) trajectories of DOK cells irradiated for 30 min, monitored between 5 and 13 h after scratching. Units in plots are in μm.

Quantification of intrinsic cell motility from individual trajectories gives complementary information that, added to collective cell migration approach, details the events. For HaCaT cells no significant impairment in the directionality of movement was noticed following UVA exposure (Figure 5A,B). The most noticeable observation was that during the first 5 h after UVA exposure, the dysplastic keratinocytes exhibited a loss in the directionality of movement. Thus, the motile ability of irradiated DOK decreased. The cells showed an undirected movement over short distances, during the first 5 h after irradiation (Figure 5D), in comparison with the mock-irradiated DOK. After longer time periods post-irradiation, cells regained their ability for long range movement to re-coat the scratched surface, although their directionality was not completely restored (Figure 5E).

The results demonstrate the transient loss in the directionality of dysplastic cells during migration as an effect of UVA irradiation. This effect is connected with the increase of the time period required by the irradiated DOK to coat the denuded surface. By comparison, in the in vitro wound healing experiments, the normal cells were not significantly affected by UVA exposure, in terms of directional movement and duration of scratch-wound closure.

### 2.3. Effects of UVA Radiation on the Actin Cytoskeleton and Focal Contacts

**Figure 6:**
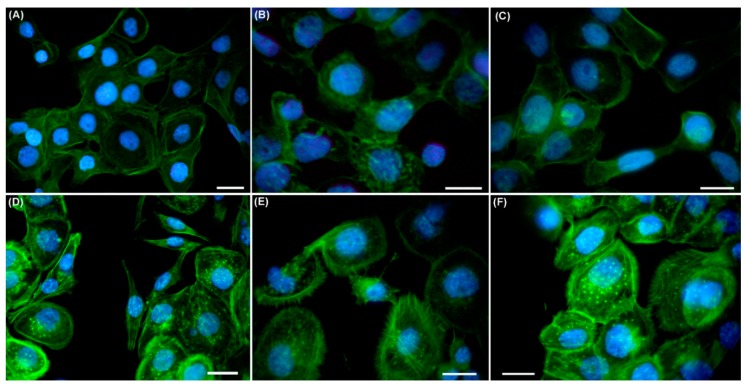
Effects of 60 min UVA exposure on actin cytoskeleton reorganization in keratinocytes. (**A**–**C**)—HaCaT; (**D**–**F**)—DOK. (**A**,**D**)—mock-irradiated cells; (**B**,**E**)—cells right after irradiation; (**C**,**F**)—cells 2 h post-irradiation. Actin filaments are marked in green; nuclei are in blue. Scale bars represent 10 μm.

Cell motion during wound closure involves repeated cycles of actin cytoskeleton-mediated protrusion, as well as cell polarization, formation of adhesive contacts, contraction and retraction at the trailing edge. In this report, changes in actin cytoskeleton dynamics as a UVA-induced response were addressed by immunofluorescence studies. Immediately after irradiation, the actin cytoskeleton showed a cortical organization, similar to that in the mock-irradiated cells, for both normal and dysplastic keratinocytes, but few stress fibers were present, mainly in DOK cells (Figure 6). Noteworthy, 2 h post-irradiation, stress fibers showed a better organization in DOK cells. Moreover, after a 60 min irradiation session, DOK cells developed many filopodia that persisted even in the cells cultured for 2 h post-irradiation, and were accompanied by the appearance of new focal contacts as compared with cells immediately after exposure (Figure 6).

**Figure 7:**
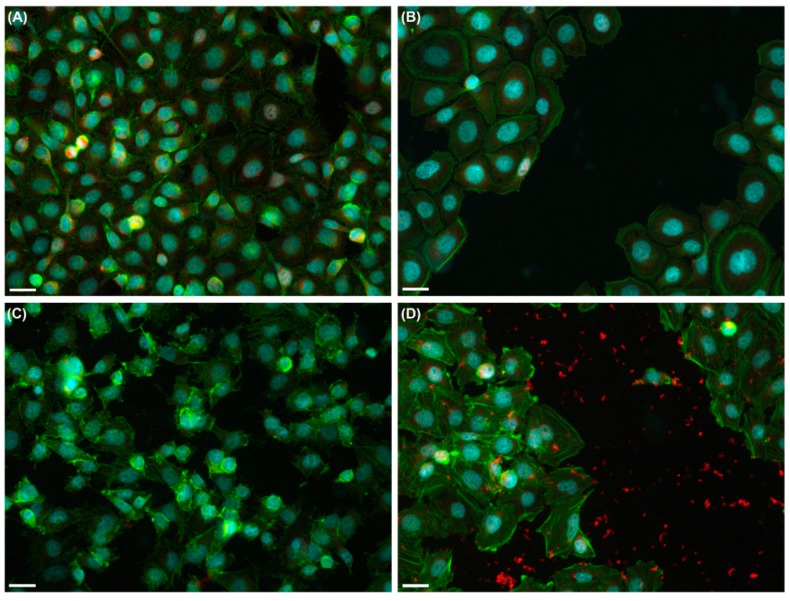
Effects of 60 min UVA exposure on the focal contacts of keratinocytes as revealed by anti-FAK immunofluorescence (in red). (**A**,**B**)—HaCaT; (**C**,**D**)—DOK. Note that UVA exposure had very few effects on normal keratinocytes (compare (**A**), for mock-irradiated cells, with (**B**) for exposed cells followed by scratching), while UVA radiation increased the strength of focal contacts in DOK cells resulting in many focal contacts extracted from the cells remaining attached to the culture surface in the scratched area. Nuclei are marked in blue with DAPI; actin filaments are in green. Scale bars represent 50 µm.

In DOK cells, the attachment to the substrate provided by focal contacts during irradiation was stronger, as evidenced by the presence of focal adhesion kinase (FAK) fluorescent signal in the denuded surface 2 h post-irradiation (Figure 7). HaCaT cells did not exhibit a stronger attachment to the substrate after irradiation.

## 3. Discussion

In this study, human keratinocyte cell lines HaCaT and DOK were used in order to investigate the effect of UVA irradiation on adhesion, spreading, proliferation and motility, including actin cytoskeleton dynamics. Although HaCaT cells are spontaneously immortalized through p53 mutation, this cell line closely approximates normal keratinocytes, providing a valuable experimental model [6,24]. The DOK line was established from a tongue dysplasia, and it is considered to have a partially transformed phenotype [25]. On the other hand, oral keratinocytes are considered to share structural and functional features with skin keratinocytes, and they offer an appropriate model of dysplastic human epithelial cells [25]. Therefore, in this study, we have employed these cell lines of normal and dysplastic keratinocytes in order to understand how UVA radiation, as an environmental stress factor on human skin, could affect the behavior of keratinocytes.

### 3.1. Cell Adhesion, Spreading, and Proliferation Are Affected by UVA Radiation

The basal keratinocytes attach to the basement membrane, and this interaction modulates their growth and differentiation, as well as the wound healing process. The basement membrane contains extracellular matrix proteins such as laminin and collagen IV. The different components of the extracellular matrix interact with cell surface receptors in a specific manner, thereby mediating the adhesion and interaction of cells with the appropriate matrix protein [26]. Collagen I is a matrix component in the dermal area that becomes available during skin wounding, while fibronectin, not present in normal epidermal basement membrane, is present at sites of wound repair [27]. An effective wound healing process depends on both cell motility and cell proliferation. We found that UVA exposure did not affect the affinity of keratinocytes for particular matrix proteins, collagen I remaining the preferred adhesion substrate for both cell types, HaCaT and DOK. Previous studies have reported altered intercellular adhesion and junctional communication in skin cells in response to UVA irradiation [10,11,28]. However, following UVA irradiation, the behavior of HaCaT and DOK cells in terms of spreading and adhesion was different, the cell index decreasing in a dose dependent manner, only for normal keratinocytes. Moreover, our results regarding cell index changes suggest that cell-matrix adhesion might also be a target for UVA radiation, affecting the proliferative ability and cell migration. The delay for cell proliferation observed in our experiments after UVA exposure, even though it is different for the two cell types investigated, would interfere with a normal repair process.

### 3.2. UVA Exposure Affects the Directionality of the Cell Movement and Might Disturb Wound Healing

The repair of a skin wound is a very complex process that is highly regulated, that includes wound detection and subsequent responses [29]. These wound responses, that are directed to restore the epithelial barrier integrity, involve both migration and proliferation of skin cells. Cell migration occurs as a spatially coordinated process, which implies a directional movement and a dynamic morphology and polarization of the migrating cells [30,31]. In the present study we found that UVA irradiation induces a disorganized, random movement of dysplastic keratinocytes, without affecting the directionality of movement in normal cells. To our knowledge, this type of UVA effect on keratinocytes was not previously reported. Taking into consideration that the directed migration of keratinocytes is critical for wound re-epithelization, our observation suggests that following UVA exposure, the normal keratinocytes could be more effective in wound healing than the dysplastic cells. This suggests that epidermis developing dysplastic keratinocytes diminishes its ability to effectively heal a wound after irradiation. Normal keratinocytes retain their spatio-temporal control of movement for wound closure, even after UVA exposure.

It is known that, in addition to its role in controlling cell cycle progression, the tumor suppressor protein p53 can also affect other cellular functions including cell migration [32]. Thus, expression of a mutant p53 can promote the loss of directionality of migration and an invasive behavior of cells [33]. DOK cells carry a partially transformed phenotype that expresses increased levels of a mutant p53, harboring a 12 bp in-frame deletion (codons 188–191) [25,34]. Thus, we may presume that there is a possible connection between this p53 mutation in DOK cells and their susceptibility to lose the directionality of movement under UVA stress. Even though HaCaT cells also carry a p53 mutation, this seems not to affect their migratory ability after UVA exposure. Our results further support the suggestion from previous studies on the different ability of various p53 mutations to affect cell motility [35].

It has been shown that the invasion of cancer cells is linked to enhanced cell motility and directional migration. Moreover, several studies have indicated that cancer cells exhibit two distinct patterns of movement into the tumor microenvironment: streaming cellular movement (coordinated cell motility), and random cellular movement (uncoordinated cell motility) [36,37], suggesting that random cell locomotion also plays an important role in tumor cell invasion [38]. Although the random migration is not a hallmark of tumor cells, the impaired directional migration, together with the increased resistance to apoptosis gained by DOK following UVA irradiation [39], suggest that the pre-malignant status acquires some new abnormal traits under UVA stress.

### 3.3. UVA Radiation Induces the Remodeling of Cytoskeleton and Adhesion Structures

In this study, we have identified changes of the actin filaments organization in the UVA-irradiated DOK cells resulting in highly organized stress fibers and the formation of filopodia, as well as new focal contacts. Focal adhesions are located along the basal surfaces of keratinocytes which are connected to actin filaments and their dynamics may mediate transitory adhesion during cell migration and wound healing. Their formation involves the recruitment and assembly of a large number of proteins, such as kinases and adaptor proteins [40]. FAK is a tyrosine kinase involved in cell migration. Signaling by FAK promotes cell motility and its inhibition leads to a defective migration [41]. FAK controls cell migration by regulating the dynamics of focal contacts [42,43,44]. At the leading edge, FAK is required for the efficient disassembly/assembly of junctions, while at the trailing edge, FAK is involved in the forward movement of the focal contacts and the retraction of the cytoplasmic tail [45]. Our results showed a higher sensitivity of DOK cells to UVA in terms of formation, dynamics and strength of focal contacts in response to UVA irradiation that affected the directionality of migration, most probably by regulating FAK activity. The enhanced strength of focal contacts (proven by the remaining contact complexes with FAK on the denuded surface after cell layer scratching) affected the distance of cell migration and the time needed for “wound healing”. In contrast with the dysplastic keratinocytes, the normal ones displayed neither significant changes of the actin cytoskeleton organization and focal contact dynamics, nor impaired directionality of migration following UVA exposure.

In conclusion, our results showed that dysplastic keratinocytes were more responsive to UVA exposure in comparison with the normal keratinocytes. Therefore, their mobility, in terms of directionality and time interval required for scratch-wound closure, was seriously affected. Moreover, UVA exposure has induced the rearrangement of actin cytoskeleton, resulting in an increased occurrence of stress fibers, filopodia and focal contacts in dysplastic keratinocytes.

## 4. Materials and Methods

### 4.1. Cell Cultures

HaCaT and DOK cells were cultured in DMEM (Dulbecco’s Modified Eagle’s Medium)/Ham F12 (Sigma-Aldrich Chemie Gmbh, Munich, Germany) and supplemented with 10% fetal bovine serum (FBS), and antibiotic-antimycotic solution (both from Lonza, Basel, Switzerland). Cells were seeded in 35 mm glass bottom petri dishes (7.5 × 10^5^ cells/dish), for time-lapse videomicroscopy wound healing experiments, or in 16 wells E-plates (7.5 × 10^3^ HaCaT cells/well, and 15 × 10^3^ DOK cells/well, respectively) for time-lapse impedance monitoring. To prepare culture surfaces for the experiments, three different matrix proteins were used: collagen type I, from calf skin (C9791, Sigma-Aldrich Chemie Gmbh); fibronectin from bovine plasma (33010-018, Invitrogen Molecular Probes, Eugene, OR, USA); and laminin from mouse basement membrane (354232, BD Bioscience, San Jose, CA, USA).

### 4.2. UVA Source and Irradiation Conditions

Irradiation was carried out with a UV-365 nm lamp, VL-340 BLB model (Vilber Lourmat, France) at a light intensity of 381 μW/cm^2^. Prior to irradiation, cells were washed twice with 2 mL phosphate buffered saline (PBS). The cell layer was covered with 2 mL PBS with Ca^2+^ and Mg^2+^ and irradiated from the top, at a distance of 10 cm. Irradiation was performed for 15, 30 or 60 min, which resulted in accumulated doses of 4.7, 9.4 and 18.7 J/cm^2^, respectively, as measured with a LaserStar Power Meter, provided with a 3A-P photodetector (Ophir Optronics Solutions Ltd., Jerusalem, Israel). To avoid thermal stimulation, UVA exposure was done in a well-ventilated laminar flow hood (Safeflow 1.8, Bioair, Siziano, Italy). The control (mock-irradiated) cells were similarly handled, but were shielded from UVA with an aluminum foil sheet. The experimental UVA doses used in our study were previously reported as low to moderate that induce adaptive, but not lethal responses in cell culture [46].

### 4.3. Cell Impedance Monitoring

The xCELLigence system (Roche Applied Science, Penzberg, Germany) was used to assess cell adherence, spreading and proliferation by time-lapse measurement of cellular layer impedance variation. This system measures the electrical impedance across a biocompatible microelectrode array, located on the bottom of plate wells. For monitoring, plates were placed in the RTCA DP (Real-Time Cell Analyzer Dual Plate) Station, inside a standard CO_2_ incubator. The equipment was connected to the controller (a laptop that collects and analyzes the data using the RTCA software). The 16-well E-Plates were coated overnight, at 4 °C, with matrix proteins (collagen, fibronectin or laminin) to three assessed concentrations 2.5, 5 or 10 µg/mL (diluted in 0.1 M NaHCO_3_). The background signal was recorded for DMEM-F12 medium, with 5% bovine serum albumin (BSA), before placing the cells inside the wells in the same medium. Cells were seeded at a density of 1.5 × 10^4^ cells/well (for DOK) and of 7.5 × 10^3^ cells/well (for HaCaT), respectively. Once the signal had reached a plateau (cells have finalized the adherence and spreading), the medium was replaced with PBS (with Ca^2+^ and Mg^2+^) prior to UVA exposure. After exposure, the buffer was replaced with DMEM-F12, supplemented with 5% BSA and 1% FBS. Time-lapse measurement of the cell layer impedance was assessed before and after UVA exposure.

### 4.4. Time-Lapse Videomicroscopy

Cell behavior and ability to re-coat the scratched surface were monitored after UVA exposure, with the BioStation IM (Nikon Corp., Amsterdam, The Netherlands). The dishes were prepared for cell seeding on the matrix protein, as detailed elsewhere [47]. After 2 h incubation at 37 °C, 5% CO_2_, for adherence and spreading, cells were irradiated for either 30 or 60 min, as detailed above. To prepare the in vitro wound healing assay, the cell monolayer was accurately scratched with an obliquely cut pipette tip, right after UVA exposure, and rinsed with PBS to remove all cellular debris [48,49], after which the PBS was replaced by the culture medium. For each experiment, 5 to 10 fields, along the scratch, were monitored until the cells succeeded in covering the denuded surface, by collecting images every 5 min. Image analysis was carried on using NIS-Elements Basic Research software (Nikon, Corp.). Cell motility was examined at the individual cell level by tracking cell migration pathways using cell centroid coordinates, automatically determined by the software after tracing the contour of the cells in the migration front. To gather the individual cell trajectories recorded by video microscopy, the so-called wind roses representations were used, by plotting the determined centroid coordinates and connecting them in the graph [48,49].

### 4.5. Immunofluorescence

Cells plated on matrix protein-coated coverslips (8 × 10^4^ cells/cm^2^) were incubated for 4 h, at 37 °C, 5% CO_2_ for adherence and spreading, followed by UVA exposure for 60 min. After irradiation, the cell layer was scratched, and cells were fixed immediately or 2 h post-irradiation at 37 °C, 5% CO_2_, followed by saponin or Triton X-100 permeabilization. Incubation with a rabbit anti-FAK (Focal Adhesion Kinase) antibody (1:50, Santa Cruz Biotech. Dallas, TX, USA) was performed overnight, at 4 °C, followed by a goat anti rabbit-Alexa Fluor^®^ 594 secondary antibody (1:400) for 1 h. Actin filaments were stained with Alexa Fluor^®^ 488-phalloidin (Invitrogen Molecular Probes), while nuclei were stained with DAPI (Sigma-Aldrich Chemie Gmbh). Samples were examined under a Nikon TE300 microscope, equipped with a Nikon DX1 camera, Nikon PlanApo 40× and 60× objectives, and the appropriate fluorescence filters.

### 4.6. Data Analysis

Data are presented as mean ± SEM of values measured for at least 5 microscopic fields/experiment in time-lapse videomicroscopy and for 4 wells in impedance monitoring for three independent experiments.

## Abbreviations

BSA	bovine serum albumin
DMEM	Dulbecco’s modified Eagle’s medium
DOK	dysplastic oral keratinocyte
FAK	focal adhesion kinase
FBS	fetal bovine serum
PBS	phosphate buffered saline
RTCA DP	real-time cell analyzer dual plate
UVA	ultraviolet A
